# The Effects of Long-Term Immunosuppressive Therapies on the Structure of the Rat Prostate

**DOI:** 10.3390/ijerph17124614

**Published:** 2020-06-26

**Authors:** Marta Grabowska, Maria Laszczyńska, Karolina Kędzierska-Kapuza, Andrzej Kram, Kamil Gill, Małgorzata Piasecka

**Affiliations:** 1 Department of Histology and Developmental Biology, Pomeranian Medical University, 71-210 Szczecin, Poland; martag@pum.edu.pl (M.G.); maria@laszczynska.pl (M.L.); kamilgill@wp.pl (K.G.); 2 Department of Nephrology, Transplantology and Internal Medicine, Pomeranian Medical University, 70-111 Szczecin, Poland; karolina.kedzierska@gmail.com; 3 Department of Pathology, West Pomeranian Oncology Center, 71-730 Szczecin, Poland; akram@onkologia.szczecin.pl

**Keywords:** Immunosuppressants, rat, ventral prostate, intermediate filaments, mast cells, plasma cells, ultrastructure, rapamycin

## Abstract

*Background:* Little is known about the overall impact of immunosuppressive drugs on the prostate. The study aimed to determine the impact of different protocols of immunosuppressive treatment on the structure of the rat ventral prostate. *Methods*: For 6 months, 48 male Wistar rats received immunosuppressive drugs: cyclosporin A, tacrolimus, mycophenolate mofetil, rapamycin, and prednisone, according to three-drug protocols. Light and transmission electron microscopic studies, and quantitative evaluation of immunohistochemical expression of selected intermediate filaments, CD117^+^ mast cells, and CD138^+^ plasma cells were performed in the rat ventral prostate. *Results:* In all experimental groups, acini focal hyperplasia, changes to the ultrastructure of the glandular epithelium, changes in the expression of cytokeratins and desmin, and numerous mast and plasma cells in the prostate stroma were found. In cyclosporine-A-based groups, atrophy and numerous intracellular vacuoles were observed. In groups where a three-drug treatment was replaced with rapamycin, morphological alterations were less severe compared to those without conversion. *Conclusions:* In the rat ventral prostate, (1) immunosuppressive protocols affect the morphology and immunohistochemical expression of intermediate filaments, (2) morphological alterations, expression, and localization of selected proteins are not connected with adenocarcinoma development, and (3) conversion of the treatment to rapamycin may prevent hyperplastic abnormalities.

## 1. Introduction

Advancements in immunosuppressive therapy have produced treatment protocols using a combination of different drugs that are clearly effective in decreasing acute rejection episodes [1]. In transplant recipients, immunosuppressive protocols are usually based on combinations of three drugs from different classes, which provide different mechanisms of action. The conventional immunosuppressive protocols include calcineurin inhibitors (e.g., cyclosporin A (CsA), tacrolimus (Tac)), corticosteroids (e.g., prednisone (Pred)), inhibitors of purine synthesis (e.g., mycophenolate mofetil (MMF)), and less routinely used mammalian target of rapamycin (mTOR) inhibitors (e.g., rapamycin (Rapa)) [2]. A combination of immunosuppressive drugs is steadily maintained within the first months after kidney transplantation. However, in some cases, modification of the immunosuppression protocols is needed because of drug toxicity. Therefore, an important factor is the choice of an appropriate immunosuppressive treatment regimen, which maximizes the overall therapeutic effectiveness and minimizes the risk of adverse effects.

As a result of the use of immunosuppressive drugs, a considerable decrease in the incidence of graft rejection has been observed; however, this has been correlated with an increased frequency of infections and cancers, including genitourinary malignancies [3,4]. One of the factors that may be important in pathomorphological diagnostics is the expression of intermediate filament (IF) proteins such as cytokeratin and desmin. Altered expression patterns related to IF that are the result of immunosuppressive drug supply may indicate that adverse changes are associated with inflammation or cancer development. Different patterns of cytokeratin expression have been observed in prostatic glandular epithelium in cases of prostate inflammation, benign prostate hyperplasia (BPH), and malignant neoplasia [5,6]. In turn, desmin is a specific marker for late-stage differentiation in the smooth muscle lineage and might be of prognostic value in evaluating patients with prostate cancer [7]. Many studies have demonstrated that administration of calcineurin inhibitors is associated with a higher risk of cancers, including prostate cancer, in patients after organ transplantation [4,8]. Despite this, little is known about the overall impact of immunosuppressive drugs on the prostate. There are only a few reports showing the influence of single immunosuppressants on this gland [9]. Our earlier research used multi-drug protocols and revealed morphological alterations and changes in the expression of specific proteins (including IF proteins and proliferating cell nuclear antigen (PCNA)), as well as abnormalities leading to the fragmentation of DNA within the rat prostate [10,11]. Until now, no other studies have reported a comparison of the long-term effects of multi-drug immunosuppressive protocols on the rat prostate. The present study is a continuation and an extension of previous research [10,11] on the rat ventral prostate. It is worth noting that the rat ventral lobe is widely used as a valuable model for the studies of human prostate diseases, and many molecular mechanisms controlling carcinogenesis and androgen-dependent processes are similar in both glands [12,13]. Moreover, the rat ventral male prostate is morphologically homologous to the female prostate (Skene’s paraurethral gland) in other rodents and humans [14].

The study was designed to determine the impact of different protocols of immunosuppressive treatment used in clinical practice, particularly on the morphology, expression of IF proteins, and presence of mast cells and plasma cells in the rat ventral prostate.

## 2. Materials and Methods 

### 2.1. Animals

**Figure 1:**
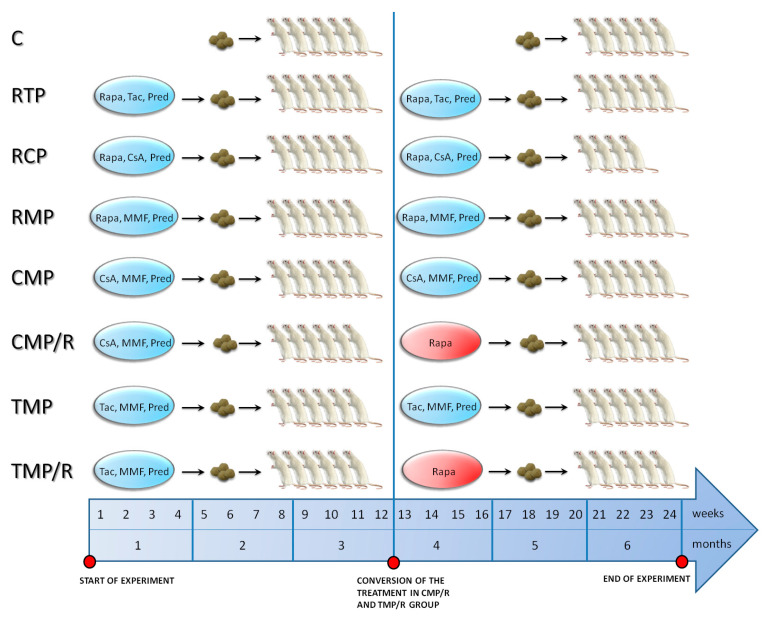
The scheme of the experiment. C: control group; CMP: rats treated with cyclosporin A, mycophenolate mofetil, and prednisone; CMP/R: rats treated with cyclosporin A, mycophenolate mofetil, and prednisone in the first three months and rapamycin in the last three months; CsA: cyclosporin A; MMF: mycophenolate mofetil; Pred: prednisone; Rapa: rapamycin; RCP: rats treated with rapamycin, cyclosporin A, and prednisone; RMP: rats treated with rapamycin, mycophenolate mofetil, and prednisone; RTP: rats treated with rapamycin, tacrolimus, and prednisone; Tac: tacrolimus; TMP: rats treated with tacrolimus, mycophenolate mofetil, and prednisone; TMP/R – rats treated with tacrolimus, mycophenolate mofetil, and prednisone in the first three months and rapamycin in the last three months.

The studies were performed on 48 sexually mature 14 week old male Wistar rats. The animals had genetic and health certificates issued by a veterinarian. Prior to the experiment, all animals survived the 2 week adaptation period. Rats were fed with the specialized laboratory diet LSM (1474 kJ/100 g; 17.6% protein (Agropol, Motycz, Poland)). The rats were divided into one control group and seven experimental groups (six rats in each group) and were housed in standard cages (six rats per cage). Rats in the control group received bread balls without any medication. The animals in the experimental groups received immunosuppressive drugs (in their pharmaceutical form) orally in a ball of bread every 24 h for 6 months. Rats were administered Rapa (0.5 mg/kg of body weight/day; Rapamune; Pfizer, Inc., New York, NY, USA), CsA (5.0 mg/kg of body weight/day; Sandimmum-Neoral; Novartis International AG, Basel, Switzerland), Tac (4.0 mg/kg of body weight/day; Prograf; Astellas Pharma Inc., Tokyo, Japan), MMF (20.0 mg/kg of body weight/day; CellCept; Hoffman-La Roche Ltd., Basel, Switzerland), and Pred (4.0 mg/kg of body weight/day; Encorton; Polfa, Pabianice, Poland), according to the standard three-drug regimens of immunosuppressive treatment commonly used in human transplant recipients (Figure 1). The drug doses were based on reported data [15,16]. In addition, they were adjusted according to body mass and were calculated to be equivalent to the doses used in human therapy, while accounting for metabolic differences. The animal welfare procedures, study design, and experimental protocol were approved by the Local Ethical Committee for Experiments on Animals of the Pomeranian Medical University, Szczecin, Poland (No. 26/2011, No. 06/08 and No. 24/08).

### 2.2. Collection of Material for the Study

Forty-six rats completed the study (two rats in the RCP group died in the fourth month of the experiment) [11]. After 6 months, all animals were anaesthetized with intraperitoneally administered ketamine hydrochloride (50 mg/kg). Ventral lobes of the rat prostates were obtained for sectioning. For immunohistochemistry, tissues were routinely fixed in 4% buffered paraformaldehyde and were then embedded in paraffin. 

### 2.3. Drug Concentrations

To determine the concentrations of individual immunosuppressive drugs, a separate experiment was performed on a group of 36 14 week old male rats. The animals were divided into one control and five experimental groups (six rats in each group). In experimental groups, rats were administered drugs every 24 h in bread balls according to the three-drug regimens and doses identical to those used in the previous experiment (except protocols with conversion to rapamycin). After two weeks, the experiment was completed. The animals were anaesthetized with ketamine hydrochloride given intraperitoneally (50 mg/kg of body mass). The immunosuppressive drugs’ concentrations were measured after 4 h of enteral administration [16]. The Rapa concentration was measured in whole blood samples collected in tubes with a 10% (*w*/*v*) sodium ethylenediamine-tetraacetic acid (EDTA) in distilled water. After centrifugation, the Rapa concentration was measured using high-performance liquid chromatography (HPLC) with the UV-absorbance method, following extraction by 1-chlorobutane. Desmethoxysirolimus constituted an internal standard. HPLC-UV analysis was performed using a Hewlett-Packard series 1050 instrument (Agilent, Palo Alto, CA, USA) with a UV variable wavelength detector. Reversed-phase chromatography was performed on an Alltech Alltima column packed with C18 (150 mm × 2.1 mm, particle size 5 μm) at 50 °C. The mobile phase was composed of acetonitrile (60%) and water (40%) (*v*/*v*), and the flow rate was 0.5 mL/min. The detection wavelength was 278 nm, and the limit of detection was 1 ng/mL. The concentrations of CsA and Tac were also measured in the whole blood of the rats, collected in tubes with a 10% (*w*/*v*) solution of EDTA in distilled water. The determination of Tac concentration was performed using an IMx test, which is based on a microparticle enzyme immunoassay (MEIA). The measurements were performed using an Abbott apparatus (Abbott Laboratories, Park, USA). The concentration of CsA was determined with an AxSYM test (Abbott Laboratories, Park, USA), which is based on a fluorescence polarization immunoassay (FPIA).

### 2.4. Transmission Electron Microscopy

The ventral lobes of the rat prostates were washed with phosphate-buffered saline (PBS) and cut into pieces of ∼1 mm^3^. Next, they were fixed with a 2.5% (*v*/*v*) glutaraldehyde solution (Sigma-Aldrich Co., St Louis, MO, USA) in 0.1 mol/L sodium cacodylate buffer (Sigma-Aldrich Co., St Louis, MO, USA) at pH 7.4 for 2 h at 4 °C. The tissue was then postfixed in 1% (*w*/*v*) osmium tetroxide (OsO4; Sigma-Aldrich Co., St Louis, MO, USA) in 0.1 mol/L cacodylate buffer for 2 h at 4 °C. The tissue was dehydrated in ethyl alcohol (30–96%) (*v*/*v*) and 100% acetone and embedded in Spurr epoxy resin (Polysciences, Inc., Warrington, PA, USA). Semi-thin sections (1 µm) and ultra-thin sections (70 nm) were cut using a Reichert OmU3 ultramicrotome (Reichert, Vienna, Austria). Before proceeding to electron-microscopic evaluation, semi-thin sections were stained with 0.4% (*w*/*v*) aqueous toluidine blue solution (Sigma-Aldrich, St. Louis, MO, USA) and examined using a light microscope (Olympus BX 41, Hamburg, Germany) to identify the area of interest for TEM. The histopathological analysis was performed in intermediate segment of rat ventral prostate according to Bosland et al. [17] guidelines. The stained sections were independently examined by two experienced pathologists. In turn, ultra-thin sections were collected on copper grids (Sigma–Aldrich, Co., St Louis, MO, USA) and double-stained with 9% alcoholic uranyl acetate (Sigma-Aldrich Co., St Louis, MO, USA) and 2.66% alkaline lead citrate (Sigma-Aldrich Co., St Louis, MO, USA). The samples were examined using a JEM-100CX TEM (JEOL, Tokyo, Japan). 

### 2.5. Immunohistochemistry

Immunostaining of paraffin-embedded ventral prostate tissue sections from rats was performed following the manufacturer’s guidelines (Dako, Glostrup, Denmark). Sections (3 μm thick) were deparaffinized with xylene and rehydrated in a graded ethyl alcohol series (99.8−50%) (*v*/*v*). Antigen retrieval was carried out by boiling the slides for 30 min in Target Retrieval Solution Citrate (Dako, Glostrup, Denmark) at pH 6.0 (for desmin) and in Target Retrieval Solution (Dako, Glostrup, Denmark) at pH 9.0 (for cytokeratin MNF116, pan-cytokeratin (CK PAN), and high-molecular-weight cytokeratins (CK HMW), CD117 and CD138). The activity of endogenous peroxidase was blocked by treating slides with peroxidase-blocking solution (Dako, Glostrup, Denmark) for 10 min. Next, the slides were washed in PBS. To determine the localization and immunoexpression of the specific intermediate proteins, the following antibodies were used: (1) rabbit monoclonal antibody IgG against CD117/c-KIT (clone EP10; Roche-Ventana Medical Systems, Tucson, AZ, USA), diluted 1:50; (2) mouse monoclonal antibody IgG against CD138/syndecan-1 (clone B-A38; Roche-Ventana Medical Systems, Tucson, AZ, USA), diluted 1:50; (3) mouse monoclonal antibody IgG against cytokeratin MNF116 (clone MNF116; Dako, Glostrup, Denmark), diluted 1:50; (4) mouse monoclonal antibody IgG cocktail against CK PAN (clone AE1/AE3/PCK26; Roche-Ventana Medical Systems, Tucson, AZ, USA), diluted 1:50; (5) mouse monoclonal antibody IgG cocktail against CK HMW (clone 34βE12; Roche-Ventana Medical Systems, Tucson, AZ, USA), diluted 1:50; and (6) mouse monoclonal antibody IgG against desmin (clone D33; Dako, Glostrup, Denmark), diluted 1:50. The sections were incubated with the primary antibodies in a humid chamber for 30 min. Afterward, the sections were incubated with a complex containing a secondary antibody conjugated with horseradish peroxidase (Dako, Glostrup, Denmark). Next, diaminobenzidine (Dako, Glostrup, Denmark) was applied. The all slides were counterstained with Mayer’s haematoxylin (Sigma-Aldrich Co., St Louis, MO, USA), dehydrated in a graded ethyl alcohol series (50−99.8%) (*v*/*v*), and xylene, and coverslipped. The slides were examined under a light microscope (Olympus BX 41, Hamburg, Germany). The controls for reaction specificity were performed by replacing the primary antibody with PBS. 

### 2.6. Quantitative Computer Image Analysis of Immunohistochemistry

**Figure 2:**
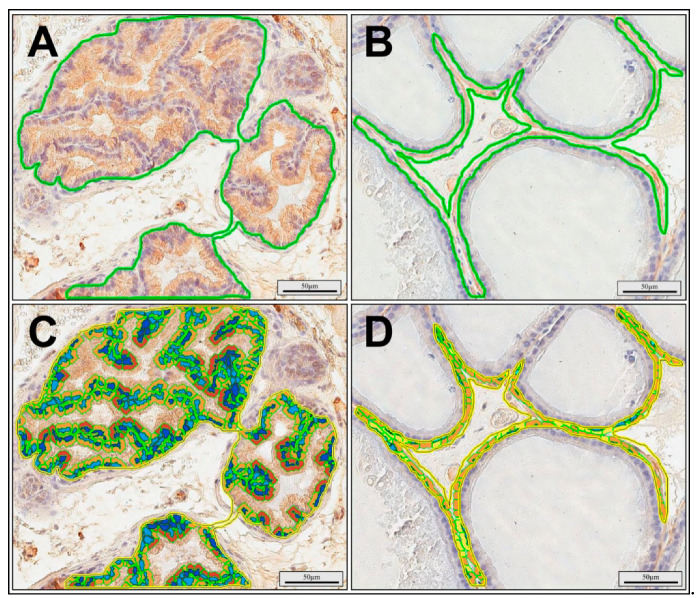
The cytoplasmic algorithm developed for the quantitative evaluation of immunohistochemical expression of cytokeratin MNF116 (**A**,**C**) and desmin (**B**,**D**) in rat ventral prostate. Determination of analysis area (**A**,**B**); marked up images with bronze, orange, and yellow pixels represent immunopositive cell cytoplasm (strong, moderate, and weak intensity, respectively), while blue pixels depict immunonegative nuclei (**C**,**D**).

Quantitative computer image analysis was performed according the manufacturer’s protocol. All slides were scanned at an absolute magnification of 200× (resolution of 0.25 μm/pixel) using a ScanScope AT2 scanner (Leica Microsystems, Wetzlar, Germany). The obtained digital images of the slides were analyzed using an ImageScope viewer (Version 11.2.0.780; Aperio Technologies, Inc., Vista, CA, USA). CD117^+^ mast cells and CD138^+^ plasma cells were manually counted in 30 random fields for each group (five random fields from each rat) with an area of 0.60 mm^2^ from the digital images of the slides. For the automatic computer analysis of cytokeratin MNF116 and desmin expression, a cytoplasmic v2 algorithm (version 11.2.0.780; Aperio Technologies, Inc.) was used. Using the software, individual stains were calibrated by analyzing sections and recording the average optical density values. The area of analysis was manually determined (Figure 2A,B). The percentage of cells with weak, medium, and strong positive immunostaining were determined for cytokeratin MNF116 (Figure 2C) and desmin (Figure 2D). The total number of cytokeratin MNF116- and desmin-positive cells was counted in 30 random fields for each group (five random fields from each rat), with an average area of 0.28 mm^2^ (for cytokeratin) and 0.12 mm^2^ (for desmin).

### 2.7. Statistical Analysis

Statistical analysis was conducted using Statistica 8.0 software (StatSoft, Krakow, Poland). For quantitative values, the Kruskal–Wallis test with Dunn’s multiple comparison test for post hoc analysis was performed. The cut-off level for statistical significance was *p* < 0.05.

## 3. Results

### 3.1. Immunosuppressive Drug Concentrations in the Blood of Rats 

The concentrations of immunosuppressive drugs (Table 1) in the blood of rats in the experimental groups were within the therapeutic range. In the blood of rats, the highest concentration of CsA (1272 ± 556.7 ng/mL) was found in the RCP group. The highest concentrations of Rapa (6.5 ± 2.4 ng/mL) and Tac (15.3 ± 9.2 ng/mL) were noted in the RTP group. The concentrations of MMF and Pred were not measured because these measurements are not used in clinical practice.

### 3.2. Light-Microscopic Studies

**Figure 3:**
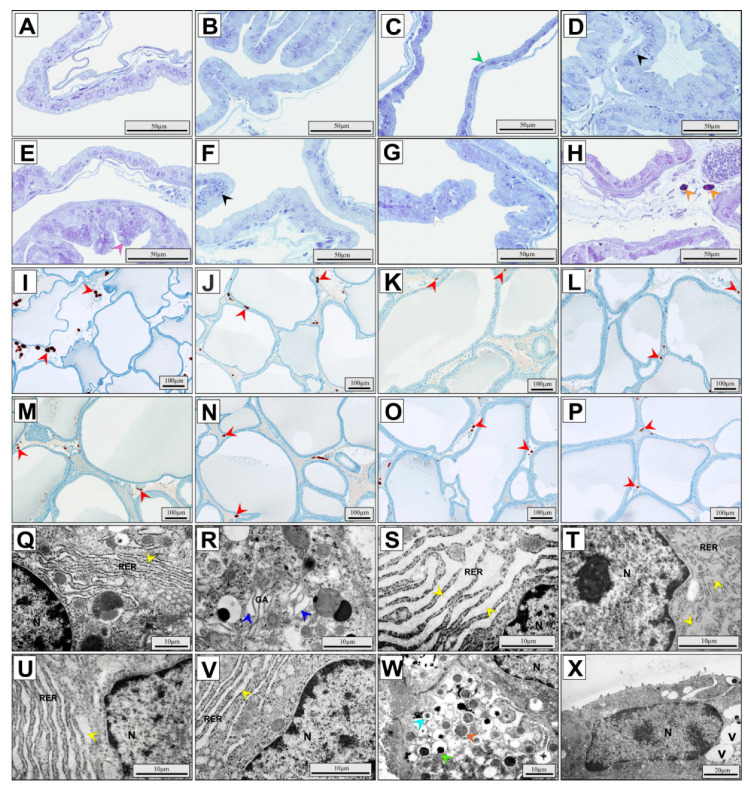
Representative light micrographs of toluidine-blue-stained glandular epithelium (**A**–**H**), immunolocalization of CD117^+^ cells in the stroma (**I**–**P**), and electron micrographs of epithelial luminal cells (**Q**–**X**) of rat ventral prostate. Normal glandular epithelium in the control group (**A**); slight changes of the epithelium in the RTP group (**B**); epithelial atrophy (green arrowhead) in the RCP group (**C**); prominent nucleolus in the nucleus (black arrowheads) and focal atypical hyperplasia (violet arrowhead) of the CMP group (**D**), the RMP group (**E**), and the TMP group (**F**); focal atypical hyperplasia without features of prostatic intraepithelial neoplasia (white arrowhead) in CMP/R group (**G**); mast cells (orange arrows) in the TMP/R group (**H**); CD117+ cells in the stroma (red arrowheads) of the CMP group (**I**), the TMP group (**J**), the control group (**K**), the RTP group (**L**), the RCP group (**M**), the RMP group (**N**), the CMP/R group (**O**), and the TMP/R group (**P**); normal cisterns of the rough endoplasmic reticulum (yellow arrowhead) in the control group (**Q**); slightly distended cisterns of the Golgi apparatus (blue arrowheads) in the RMP group (**R**); distended cisterns of the rough endoplasmic reticulum (yellow arrowheads) in the CMP group (**S**), the TMP group (**T**), the CMP/R group (**U**), and the TMP/R group (**V**); condensed (light green arrowhead), flocculent (brown arrowhead), and star-shaped (light blue arrowhead) secretions in the RTP group (**W**); epithelial atrophy and intracellular vacuoles in the RCP group (**X**). CMP: rats treated with cyclosporin A, mycophenolate mofetil, and prednisone; CMP/R: rats treated with cyclosporin A, mycophenolate mofetil, and prednisone in the first three months and rapamycin in the last three months; GA: Golgi apparatus; N: nucleus; RCP: rats treated with rapamycin, cyclosporin A, and prednisone; RER: rough endoplasmic reticulum; RMP: rats treated with rapamycin, mycophenolate mofetil, and prednisone; RTP: rats treated with rapamycin, tacrolimus, and prednisone; TMP: rats treated with tacrolimus, mycophenolate mofetil, and prednisone; TMP/R: rats treated with tacrolimus, mycophenolate mofetil, and prednisone in the first three months and rapamycin in the last three months; v: vesicle.

**Figure 4:**
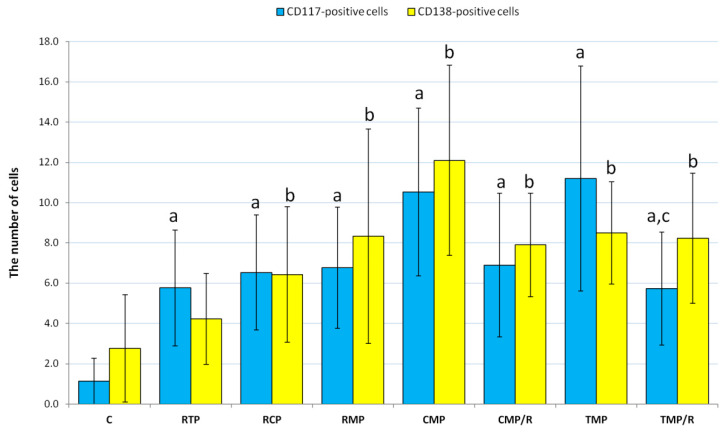
The average number of CD117^+^ and CD138^+^ cells in the stroma of rat ventral prostates in the control and experimental groups. C: control group; CMP: rats treated with cyclosporin A, mycophenolate mofetil, and prednisone; CMP/R: rats treated with cyclosporin A, mycophenolate mofetil, and prednisone in the first three months and rapamycin in the last three months; RCP: rats treated with rapamycin, cyclosporin A, and prednisone; RMP: rats treated with rapamycin, mycophenolate mofetil, and prednisone; RTP: rats treated with rapamycin, tacrolimus, and prednisone; TMP: rats treated with tacrolimus, mycophenolate mofetil, and prednisone; TMP/R: rats treated with tacrolimus, mycophenolate mofetil, and prednisone in the first three months and rapamycin in the last three months; a, b: *p* < 0.001 vs. control; c: *p* < 0.05 vs. TMP (Kruskal-Wallis test, *n* = 30 for each group); error bars represent standard deviations.

Before proceeding electron-microscopic evaluation, semi-thin sections stained with toluidine blue solution were examined (Figure 3). The rat ventral prostate has a tubulo-acinar structure. In the control and experimental groups, rats’ acini were lined with cylindrical or cubical glandular epithelium, in which both luminal and basal cells were observed (Figure 3A–H). Fibrous connective tissue, blood vessels, smooth muscle fibers, and nerve tissue were observed in the stroma. The epithelium of rat ventral prostate in the control group exhibited a relatively normal structure (Figure 3A). In all experimental groups (RTP, RCP, RMP, CMP, CMP/R, TMP, and TMP/R), adverse morphological alterations in glandular epithelium were found; however, the smallest changes were observed in the RTP group (Figure 3B). Atrophy was mainly observed in the epithelium of the RCP group (Figure 3C), and in only a few areas in the CMP group. Focal atypical hyperplasia was mainly found in the CMP (Figure 3D) and TMP groups, but there were a few similar observations in the RMP (Figure 3E), CMP/R, and TMP/R groups. In the RMP group, reactive hyperplasia in association with inflammatory cell infiltrate was also noted. Focal atypical hyperplasia was manifested by the proliferation of luminal epithelial cells forming papillary infoldings protruding into the lumen of acini, which do not obliterate the lumen of acini. Atypical cells often had increased cytoplasmic/nuclear ratios and slightly hyperchromatic nuclei. In some areas, the glandular epithelium was multilayered, and stratification of cell nuclei was noted. Moreover, in the CMP and TMP groups, cytological alterations in the form of thick nuclear envelope, prominent nucleolus in the nucleus, and increased variability in nuclear size were regularly observed (Figure 3D,F). These alterations constitute part of the criteria for low-grade prostatic intraepithelial neoplasia (PIN). Interestingly, in the CMP/R and TMP/R groups, focal atypical hyperplasia was less severe (Figure 3G,H) compared to those in the CMP and TMP groups. Additionally, in the CMP and TMP groups, the average number of CD117^+^ mast cells (10.5 and 11.2 respectively; Figure 3I,J) and CD138^+^ plasma cells (12.1 and 8.5 respectively) was higher than that in control (Figure 3K) and all other experimental groups (Figure 3L–P and Figure 4). In the CMP/R and TMP/R groups, the average number of CD117^+^ and CD138^+^ cells was lower (statistically insignificant vs. CMP group, *p* = 0.005 vs. TMP group for CD117^+^ and statistically insignificant for CD138^+^) compare to those in the CMP and TMP groups.

### 3.3. Transmission-Electron-Microscopic Studies

The luminal cells were the most abundant cells in the glandular epithelium of the ventral prostate in control rats. These cells were mostly tall columnar cells with basal euchromatic nuclei. Numerous microvilli as well as minute cytoplasmic projections on the apical surface of these cells were observed. The chromatin substructure was not changed. Normal numbers and structure of mitochondria were observed. Proper cisterns of the rough endoplasmic reticulum (RER) in the perinuclear region (Figure 3Q) and normal Golgi cisterns in the apical part of the luminal cells were observed. Many secretory vesicles were also found. In turn, basal cells with large flat nuclei were revealed. In all experimental groups (RTP, RCP, RMP, CMP, CMP/R, TMP, TMP/R), ultrastructural changes in luminal cells of glandular epithelium of ventral prostate were noted mainly in the RER and the Golgi apparatus (GA). Cisterns of the RER and the GA were distended (Figure 3R–V). In these cells, few mitochondria, cytoplasmic vacuoles, or secretory vesicles were observed. In some areas, the epithelium showed stratification—most cells were of variable size. Additionally, in the RMP group, disorders in the organization of organelles and shrunken irregular nuclei were noticed; however, distended cisterns of the GA in the luminal cells were rarely observed (Figure 3R). In the CMP and TMP groups, some areas of the epithelium had abnormal ultrastructure, and the cells had distended cisterns of the RER and the GA as well as clearly defined nuclei, a prominent nucleolus in the nucleus, and a thickened nuclear envelope (Figure 3S,T). In the CMP/R and TMP/R groups, changes in the ultrastructure of luminal cells were less severe (Figure 3U,V) than they were in the CMP and TMP groups, respectively. Interestingly, in only a few regions of the epithelium of the RTP group, luminal cells were characterized by distended cisterns of the RER and the GA. In this group, many secretory vesicles with condensed, flocculent, and star-shaped secretions were found (Figure 3W). It is worth noting that in the CsA-based groups (RCP, CMP), numerous intracellular vacuoles were observed (Figure 3X). In the RCP group and, to a lesser extent, in selected areas of the CMP group, epithelial atrophy, reduction of secretory organelles, and lysosomal vesicles were found (Figure 3X).

### 3.4. Immunolocalization of Cytokeratin and Desmin

**Figure 5:**
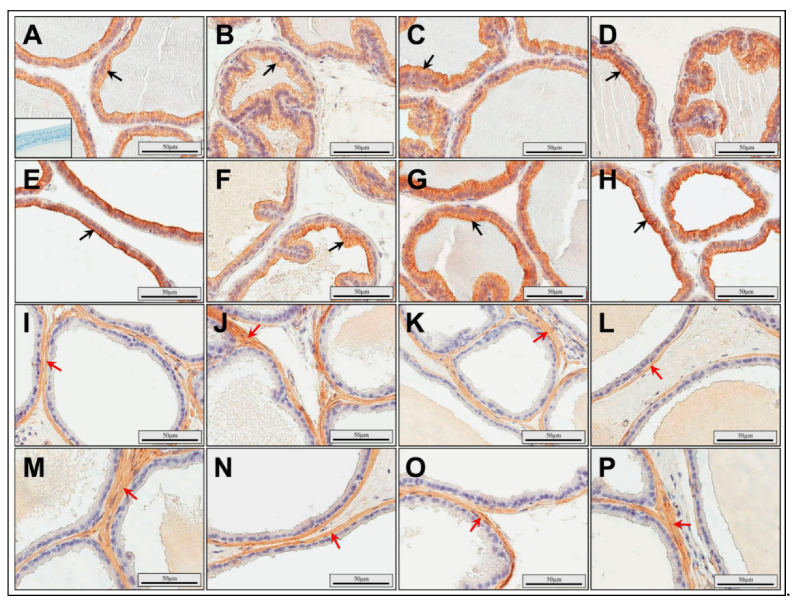
Representative light micrographs of immunolocalization of cytokeratin MNF116 (**A**–**H**) and desmin (**I**–**P**) in the rat ventral prostate. The different immunoexpression of cytokeratin in the cytoplasm (brown color) of epithelial cells of acini (black arrows) and desmin in the cytoplasm (brown color) of smooth myocytes around acini (red arrows) in the control group (**A**,**I**), the RTP group (**B**,**J**), the RCP group (**C**,**K**), the RMP group (**D**,**L**), the CMP group (**E**,**M**), the CMP/R group (**F**,**N**), the TMP group (**G**,**O**), and the TMP/R group (**H**,**P**); The inset shows a negative control (**A**). CMP: rats treated with cyclosporin A, mycophenolate mofetil, and prednisone; CMP/R: rats treated with cyclosporin A, mycophenolate mofetil, and prednisone in the first three months and rapamycin in the last three months; RCP: rats treated with rapamycin, cyclosporin A, and prednisone; RMP: rats treated with rapamycin, mycophenolate mofetil, and prednisone; RTP: rats treated with rapamycin, tacrolimus, and prednisone; TMP: rats treated with tacrolimus, mycophenolate mofetil, and prednisone; TMP/R: rats treated with tacrolimus, mycophenolate mofetil, and prednisone in the first three months and rapamycin in the last three months.

Cytokeratin MNF116-positive luminal and basal cells were observed in the glandular epithelium of the rat ventral prostate in the control and experimental groups. The cells showing immunoexpression of cytokeratin were characterized by a brown-stained cytoplasm (Figure 5A–H). To exclude cancerous alterations in rat ventral prostate in all experimental groups, immunolocalization of CK PAN and CK HMW was determined. In all groups, CK PAN and CK HMW expression was observed in luminal and basal epithelial cells of the glandular epithelium, respectively. Desmin-positive smooth muscle cells with brown-stained cytoplasm were found around the acini in the stroma (Figure 5I–P).

### 3.5. Quantitative Evaluation of Cytokeratin MNF116 and Desmin Immunoexpression

**Figure 6:**
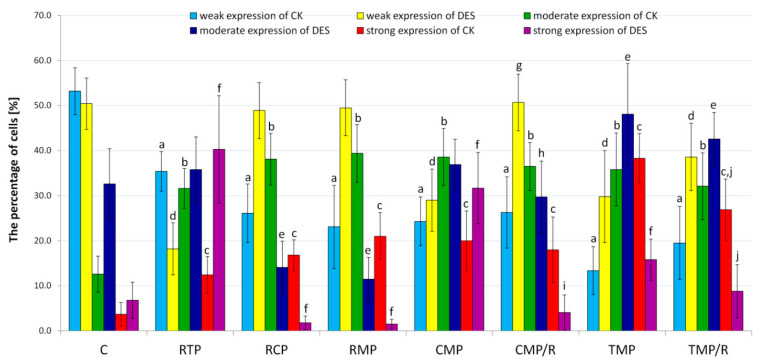
The percentage of cytokeratin- and desmin-positive cells in the rat ventral prostate. C: control group; CK: cytokeratin; CMP: rats treated with cyclosporin A, mycophenolate mofetil, and prednisone; CMP/R: rats treated with cyclosporin A, mycophenolate mofetil, and prednisone in the first three months and rapamycin in the last three months; DES: desmin; RCP: rats treated with rapamycin, cyclosporin A, and prednisone; RMP: rats treated with rapamycin, mycophenolate mofetil, and prednisone; RTP: rats treated with rapamycin, tacrolimus, and prednisone; TMP: rats treated with tacrolimus, mycophenolate mofetil, and prednisone; TMP/R: rats treated with tacrolimus, mycophenolate mofetil, and prednisone in the first three months and rapamycin in the last three months; *p* < 0.001 vs. control for weak (a), moderate (b), and strong (c) expression of cytokeratin; *p* < 0.001 vs. control for weak (d), moderate (e), and strong (f) expression of desmin; *p* < 0.05 vs. CMP for weak (g), moderate (h), and strong (i) expression; *p* < 0.05 vs. TMP for strong (j) expression (Kruskal–Wallis test, *n* = 30 for each grade of expression in each group); error bars represent standard deviations.

The percentages of cytokeratin MNF116-positive cells with weak, moderate, and strong expression (Figure 6, Table S1) in the control group differed statistically (*p* < 0.001) from what was observed in all of the experimental groups. The highest percentages of cytokeratin MNF116-positive cells with weak expression were noted in the control group (53.2 ± 5.2%) and in the RTP group (35.4 ± 4.4%), while the lowest percentage was noted in the TMP group (13.4 ± 5.3%). The highest percentage of cytokeratin MNF116-positive cells with moderate expression was found in the RMP group (39.4 ± 6.4%), while the lowest percentages were found in the control group (12.6 ± 4.0%) and in the RTP group (31.6 ± 4.4%). The highest percentage of cytokeratin MNF116-positive cells with strong expression was found in the TMP group (38.3 ± 5.5%), while the lowest percentages were found in the control group (3.7 ± 2.6%) and in the RTP group (12.4 ± 4.1%). In the CMP/R group, the percentages of cells showing moderate (36.5 ± 5.3%) and strong (18.0 ± 7.3%) expression of cytokeratin MNF116 were lower than they were in the CMP group (38.6 ± 6.3% and 20.0 ± 6.6%, respectively), but the differences were not statistically significant. Similarly, in the TMP/R group, the percentages of cells showing moderate (32.1 ± 7.4%) and strong (26.9 ± 6.8%) expression of cytokeratin MNF116 were lower than those in the TMP group (35.8 ± 8.1% and 38.3 ± 5.5%, respectively). In these cases, the difference in cells with moderate expression between the groups was insignificant, but the difference in cells with strong expression between the groups was significant (*p* = 0.031).

The percentage of desmin-positive cells (Figure 6, Table S1) with weak expression in the control group differed statistically (*p* < 0.001) from that in the RTP, CMP, TMP, and TMP/R groups. The highest percentage of desmin-positive cells with weak expression was found in the CMP/R group (50.7 ± 6.3%), while the lowest percentage was found in the RTP group (31.9 ± 7.9%). The percentage of desmin-positive cells with moderate expression in the control group differed statistically (*p* < 0.001) from that in the RCP, RMP, TMP, and TMP/R groups. The highest percentage of desmin-positive cells with moderate expression was found in the TMP group (48.1 ± 11.2%), while the lowest percentage was found in the RMP group (11.5 ± 4.8%). The percentage of desmin-positive cells with strong expression in the control group differed statistically (*p* < 0.001) from that in the RTP, CMP, and TMP groups. The highest percentage of desmin-positive cells with strong expression was observed in the RTP group (21.6 ± 9.4%), while the lowest percentage was observed in the CMP/R group (4.1 ± 3.9%). In the CMP/R group, the percentages of cells showing moderate (29.7 ± 8.0%) and strong (4.1 ± 3.9%) expression of desmin were lower than those in the CMP group (36.9 ± 5.6% and 20.0 ± 8.9%, respectively). The differences in the values of cells with moderate and strong expression among the groups were significant (*p* = 0.007 and *p* < 0.001, respectively). Similarly, in the TMP/R group, the percentages of cells showing moderate (42.6 ± 5.9%) and strong (8.8 ± 5.9%) desmin expression were lower than those in the TMP group (48.1 ± 11.2% and 15.8 ± 4.6%, respectively). In these cases, the difference in the values of cells with moderate expression was insignificant among the groups, but the difference in the values of cells with strong expression was significant among the groups (*p* = 0.037).

## 4. Discussion

Immunosuppressive drugs are crucial for improving the survival of patients after organ transplantation. However, long-term treatment with immunosuppressive drugs may also contribute to an increased risk of particular organ damage and the occurrence of inflammation and malignancies within the prostate.

**Figure 7:**
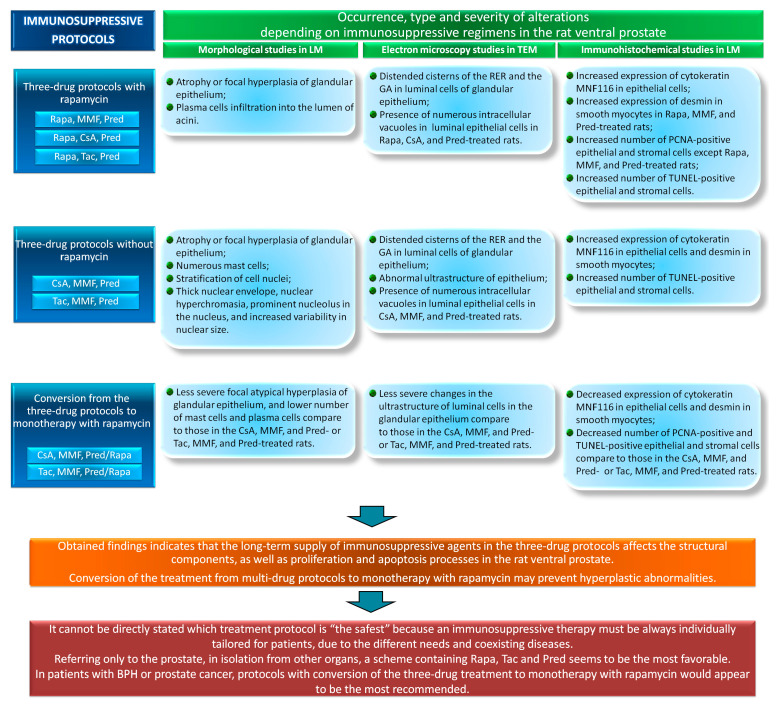
The effects of immunosuppressive regimens on the rat ventral prostate based on current and previous [10] studies (details in the text). BPH: benign prostate hyperplasia; CsA: cyclosporin A; GA: Golgi apparatus; LM: light microscopy; MMF: mycophenolate mofetil; PCNA: proliferating cell nuclear antigen; Pred: prednisone; Rapa: rapamycin; RER: rough endoplasmic reticulum; Tac: tacrolimus; TEM: transmission electron microscopy; TUNEL: terminal deoxynucleotidyl transferase-mediated dUTP nick-end labeling.

The present study is a continuation and an extension of research concerning the influence of different immunosuppressive regimens on the rat prostate [10,11]. Our earlier research indicated that long-term supply of the immunosuppressive agents in three-drug protocols affected the structural components, proliferation and apoptosis processes in the rat ventral and dorso-lateral prostate [10,11]. The present study confirmed this and presents in detail alterations in the ventral prostate (Figure 7), and also revealed the existence of an association between immunosuppressive regimens, ultrastructural alterations, IFs, number of mast cells and plasma cells, and carcinogenesis processes in a rat ventral lobe. Additionally, we have summarized knowledge about the effects of immunosuppressive drugs on the rat’s ventral prostate. 

It should be noted that many studies have addressed the effects of single immunosuppressants in different organs and tissues. However, immunosuppressive agents may interact and affect each other’s metabolism and action [18]. In addition, the effects of multi-drug immunosuppressive protocols usually used in humans may differ significantly from those obtained for single-drug regimens. In the present study, for the first time, ultrastructural changes and shifts in the expression of selected cytoskeleton proteins in the rat ventral prostate were analyzed to assess multi-drug protocols as well as the conversion of three-drug treatment to Rapa monotherapy. In order to eliminate the factors interfering with the picture of the immunosuppressive drugs action, such as the ischaemia-reperfusion periods, comorbid diseases, or immunological elements associated with the tissue compatibility antigens, the experiment was conducted in rats in which transplantation was not performed. In our research, we applied a model of therapy that is comparable to the long-term immunosuppressive treatment used in a clinical practice in humans following organ transplantation. The duration of immunosuppressive drug administration was 6 months, which is analogous to approximately 15 years of human life [19]. It is worth noting that many researchers have applied a relatively short duration of immunosuppressive agent administration [9,15]. In our study, animals received drugs orally, while in other studies, immunosuppressive drugs were administered intraperitoneally, subcutaneously, or intravenously [20,21].

The applied doses of immunosuppressive drugs allowed us to obtain drug concentrations in the blood of the animals within the therapeutic range, rather than the toxic levels seen in other studies [22,23]. In the experiment performed by Rovira et al. [22], 12 h after the intraperitoneal administration of Rapa, the concentration reached 38 ng/ml. In our study, 4 h after oral administration of Rapa, the concentrations were appreciably lower. We decided to measure drug concentrations in rats 4 h after enteral administration due to the metabolism of drugs being faster in rats than in humans [16].

Light- and transmission-electron-microscopic evaluation showed that the RTP group had the smallest alterations in the glandular epithelium of the rat ventral prostate. In our earlier reports, we made similar observations through histological evaluation [10,11]. In groups in which Rapa, CsA, and Pred as well as CsA, MMF, and Pred were administered, epithelial atrophy and a reduction in the secretory organelles in the rat ventral prostate were found. All transmission-electron-microscopic findings were consistent with the alterations observed in the semi-thin sections. In our previous studies on the rat dorsolateral prostate [11], we found that in rats that received CsA, MMF, and Pred, the epithelium was also markedly atrophic, while in the rat ventral prostate [10], morphological evaluation using a light microscope revealed a distinctly atrophic epithelium in Rapa, CsA, and Pred-treated rats. Other researchers confirmed that CsA administration may lead to epithelial atrophy. Freitas et al. [9] observed atrophy of the epithelium following a reduction in RER and GA or their total absence in the ventral prostate of CsA-treated rats. 

In the present study, CsA-based protocols contributed to the occurrence of numerous intracellular vacuoles. Studies from other authors performed on cell cultures of human T lymphocytes, monocytes, and PtK2 cells revealed that CsA induced intracellular vacuoles and nuclear lobules. These specific alterations have been attributed to the interactions of CsA with IFs [24,25]. In our study, in groups in which conversion of the treatment from CsA- and Tac-based protocols to monotherapy with Rapa occurred, there was less focal atypical hyperplasia and fewer alterations of luminal cell ultrastructure than was observed in the CMP and TMP groups. These findings may be related to a decrease in PCNA-positive and TUNEL-positive cells in our previous research on the rat ventral prostate [10]. Other authors [26] have confirmed that Rapa decreases focal hyperplasia and prevents hypertrophic and hyperplastic alterations in sulpiride-induced BPH in rats.

In our experiment involving CsA, MMF, and Pred or Tac, MMF, and Pred treatment of rats, numerous mastocytes and plasma cells were noted. It is known that mast cells participate in the induction and pathogenesis of selected inflammatory diseases. Previous research revealed that infiltrating inflammatory cells play a crucial role in BPH development [27]. Therefore, a significant increase in mast cell number and plasma cell infiltration in selected experimental groups could imply a potential role for these cells in inflammation progression or enhancing the development of BPH.

In our study, it was shown that selected combinations of immunosuppressive drugs affected immunohistochemical IF patterns in the epithelium of rat ventral prostates. The highest percentage of cytokeratin MNF116-positive cells with strong expression was found in the groups in which Tac (TMP and TMP/R) was administered. In turn, the CsA, MMF, and Pred or the Rapa, Tac, and Pred treatments of rats produced the highest percentage of desmin-positive cells with strong expression. In our recent studies on the rat dorsolateral prostate [11], we obtained similar results. Unfortunately, no literature reports regarding the expression of cytokeratin and desmin in the prostate as a result of the use of immunosuppressive drugs were found. However, Rezzani et al. [28] demonstrated that CsA induced an increase in the number of IFs and induced modifications to desmin filaments. 

It is known that most epithelial tumors maintain a specific cytokeratin expression that is associated with the origin of cells. Therefore, cytokeratins are extensively used as immunohistochemical markers in diagnostic pathomorphology. Cytokeratin PAN can be used as a marker of normal and abnormal epithelial cells, and also to determine the lineage of poorly differentiated malignant tumors [5]. In turn, CK HMW are expressed in various normal and neoplastic epithelial cells. It has been revealed that CK HMW in the prostate are markers of basal epithelial cells in normal glands. Prostatic adenocarcinoma, which is an invasive form of cancer, typically lacks a basal cell layer, meaning there is no expression of CK HMW [6]. In our study, both CK PAN and CK HMW positive expression and morphological observations allowed us to exclude prostate adenocarcinoma.

In our research into some areas of epithelium, the observed changes in selected experimental groups met some of the criteria of low-grade PIN. Indirectly, this may be related to alterations in cytokeratin expression. Yang et al. [29] revealed that cytokeratin 19 was expressed in luminal cells at high levels in PIN lesions. The available data indicate a selective adverse effect of CsA on cytokeratin filaments in other models [25,30]. Studies performed on the cell line PtK2 show that after 24 h of exposure to CsA [25], CK filaments were thicker and had decreased attachment to the plasma membrane compared to what was observed in control cells. Other researchers [30] have noted that CsA affects human keratinocytes in culture by blocking the formation of cytoskeletal tonofibrils. 

It has been suggested that CsA affects the molecular structure of the cytoskeletal elements via hydrophobic interactions, influence on the cytokeratin regulatory system, or by binding to transmembrane receptors responsible for rearrangement of intracellular components. In effect, CsA may induce depolymerization of cytoskeletal proteins and modulate selected cellular processes [28]. The mechanism of action of CsA is based on its specific binding to cyclophilin A. This complex inhibits the action of calcineurin (CaN), which is Ca^2+^/calmodulin-dependent protein phosphatase, involved in the activation of nuclear factor of activated T cell (NFATc) proteins. In normal conditions, activation of T-cell receptors leads to increase of Ca^2+^ and subsequent activation of CaN, which causes dephosphorylation of NFATc, thereby facilitating its translocation to the nucleus and regulation of gene expression [31]. It is known that CaN directly controls cytoskeleton organization via NFATc-dependent transcriptional effects and also assembly of microtubules [32]. Therefore, it may be suspected that Tac, which shows a mechanism of action analogous to that of CsA by inhibiting CaN, interferes with the control of cytoskeletal filament organization. Our research revealed that conversion of treatment from a three-drug protocol to monotherapy with Rapa caused a decrease in the expression of cytokeratin MNF116 and desmin. We suggest that a decrease in IF expression may be an effect of the antiproliferative properties of Rapa. Published data [33] show that Rapa inhibits vascular smooth muscle cell migration and proliferation by blocking the progression of the cell cycle. 

It is known that long-term use of immunosuppressive drugs may have a negative effect on male fertility. Previously, research has revealed that immunosuppressants may cause testicular injury and alterations to the hypothalamic–pituitary–gonadal axis [34]. However, it should be noted that the prostate plays an important role in male fertility. In both young and aged men, disorders in prostate structure and function might affect the functioning of spermatozoa and, consequently, fertility [35]. Our results demonstrated the adverse impact of selected treatment protocols on the prostate (Figure 4). Previously published data revealed a relationship between immunosuppressive protocols and the development of prostate cancer [4]. Therefore, determination of the cytokeratin and desmin expression patterns might become the basis for new pathophysiological diagnostic tools adapted to individual immunosuppressive treatment protocols. In addition, there is a need for further research in these directions for the prostate.

Our research has limitations. Antibodies against cytokeratin-MNF116-labeled cytokeratins 5, 6, 8, and 17 were used. According to the literature reports, in adult rat ventral prostate, in luminal epithelial cells, the expression of cytokeratins 8, 18, and 19 was detected, while in basal epithelial cells, the expression of cytokeratins 5, 7, 14, and 19 was found [36]. Therefore, it can be stated that in our study in luminal cells, expression of cytokeratin 8 was observed, and cytokeratin 5 expression was noted in basal cells. It is worth mentioning that relating animal data to humans should be undertaken with special care, because interspecies differences in the anatomical structure of the prostate and in drug metabolism exist.

## 5. Conclusions

In summary, our research provides new knowledge on the effects of immunosuppressive treatment on the rat ventral prostate. In conclusion, we demonstrated that long-term supply of immunosuppressive drugs in multi-drug protocols affects the morphology of luminal cells of the glandular epithelium and immunohistochemical expression patterns of IFs in epithelial and stromal cells of the rat ventral prostate. It can be stated that CsA-based protocols might be a cause of the occurrence of numerous intracellular vacuoles and epithelial atrophy. Moreover, in selected experimental groups, the morphological changes and the immunohistochemical localization of selected proteins were not connected with the development of adenocarcinoma; these changes are probably a result of the chronic inflammatory process. It should be highlighted that conversion of the treatment from CsA- and Tac-based protocols to Rapa may prevent hyperplastic abnormalities in the rat ventral prostate. The obtained findings justify the clinical use of mTOR inhibitors in therapy in patients with cancers, including transplant recipients suffering from prostate cancer. Moreover, Rapa may be used for therapy in men with BPH after transplantation.

Based on the obtained results, it cannot be directly stated which treatment regimen is the best for patients after transplantation, because the effects of immunosuppressive drugs on other organs should also be considered. However, when referring only to the prostate, a regimen containing Rapa, Tac, and Pred seems to be less dangerous. The obtained results might be useful in the explanation the underlying mechanisms of prostate diseases under immunosuppression. Moreover, detailed analysis, proper linking of selected processes, and understanding of interspecies differences and the limitations of the rat model may allow the obtained data to be fitted to humans.

## Figures and Tables

**Table 1:** Drug concentrations (ng/mL) in the blood of rats in the experimental groups.

Group	Cyclosporin A		Tacrolimus		Rapamycin	
	Median (Range)	X ± SD	Median (Range)	X ± SD	Median (Range)	X ± SD
RTP	−	−	12.4 (6.8−28.2)	15.3 ± 9.2	5.6 (4.6−10.5)	6.5 ± 2.4
RCP	952.5 (887.0−2149.0)	1272.0 ± 556.7	−	−	5.2 (3.0−15.0)	6.3 ± 4.4
RMP	−	−	−	−	1.2 (1.0−7.3)	2.3 ± 2.1
CMP	818.0 (647.9−865.2)	785.2 ± 83.3	−	−	−	−
TMP	−	−	7.7 (1.5−30.5)	14.1 ± 13.1	−	−

CMP: rats treated with cyclosporin A, mycophenolate mofetil, and prednisone; RCP: rats treated with rapamycin, cyclosporin A, and prednisone; RMP: rats treated with rapamycin, mycophenolate mofetil, and prednisone; RTP: rats treated with rapamycin, tacrolimus, and prednisone; TMP: rats treated with tacrolimus, mycophenolate mofetil, and prednisone; X ± SD: arithmetical mean ± standard deviation.

## Supplementary Materials

Supplementary materials can be accessed at: https://www.mdpi.com/1660-4601/17/12/4614/s1. Table S1: The percentage of cytokeratin MNF116-positive epithelial cells and desmin-positive smooth myocytes around the acini in a rat ventral prostate.
