# Supplementary material for: The Effects of Long-Term Immunosuppressive Therapies on the Structure of the Rat Prostate
Source: Int J Environ Res Public Health. 2020 Jun 26;17(12):4614. doi: 10.3390/ijerph17124614 (PMC7345872; doi:10.3390/ijerph17124614)
Supplement: Supplementary file 1 [file ijerph-17-04614-s001.pdf]

Supplementary Material

Table S1. The percentage of cytokeratin MNF116-positive epithelial cells and desmin-positive smooth myocytes around the acini in a rat ventral prostate.

Group	Intermediate Fibers	Cells with Weak Expression (%)		Cells with Moderate Expression (%)		Cells with Strong Expression (%)	
		Median (range)	X ± SD	Median (range)	X ± SD	Median (range)	X ± SD
Control	CK	53.2 (40.3–65.5)	53.2 ± 5.2	12.2 (6.6–20.0)	12.6 ± 4.0	3.0 (1.1–11.3)	3.7 ± 2.6
	DES	50.1 (40.8–64.0)	50.4 ± 5.7	32.7 (17.9–45.1)	32.6 ± 7.8	5.8 (1.7–20.2)	6.8 ± 4.0
RTP	CK	35.7 (25.7–45.7)	35.4[a] ± 4.4	32.4 (23.4–39.6)	31.6[a] ± 4.4	12.5 (5.3–18.6)	12.4[a] ± 4.1
	DES	31.8 (19.0–47.6)	31.9[a] ± 7.9	35.4 (22.5–57.4)	35.8 ± 7.2	20.6 (6.1–38.7)	21.6[a] ± 9.4
RCP	CK	26.8 (6.9–38.0)	26.1[a] ± 6.5	39.0 (21.7–46.9)	38.1[a] ± 5.7	18.3 (7.5–22.4)	16.8[a] ± 3.4
	DES	49.7 (30.9–63.4)	48.9 ± 6.2	12.2 (6.6–26.8)	14.1[a] ± 5.8	5.6 (2.9–8.1)	5.7 ± 1.2
RMP	CK	20.7 (13.1–41.4)	23.1[a] ± 9.2	39.4 (12.0–51.0)	39.4[a] ± 6.4	21.1 (12.0–36.4)	21.0[a] ± 5.2
	DES	49.8 (37.2–63.0)	49.5 ± 6.2	11.9 (3.8–24.4)	11.5[a] ± 4.8	5.8 (1.5–7.9)	5.6 ± 1.6
CMP	CK	25.3 (11.7–34.6)	24.3[a] ± 5.4	41.4 (24.2–46.7)	38.6[a] ± 6.3	21.4 (9.8–35.6)	20.0[a] ± 6.6
	DES	30.6 (23.3–50.3)	32.4[a] ± 6.7	38.2 (18.4–44.3)	36.9 ± 5.6	16.1 (10.4–33.6)	20.0[a] ± 8.9
CMP/R	CK	27.5 (8.7–37.7)	26.3[a] ± 7.9	33.4 (30.6–44.0)	36.5[a] ± 5.3	17.2 (3.2–34.8)	18.0[a] ± 7.3
	DES	52.0 (34.6–66.9)	50.7 ± 6.3	31.0 (6.4–39.2)	29.7[b] ± 8.0	2.8 (0.8–22.1)	4.1[b] ± 3.9
TMP	CK	11.8 (7.0–28.8)	13.4[a] ± 5.3	34.8 (20.6–53.1)	35.8[a] ± 8.1	40.3 (30.5–52.9)	38.3[a] ± 5.5
	DES	28.9 (8.3–50.7)	29.8[a] ± 10.2	48.2 (24.7–68.0)	48.1[a] ± 11.2	15.0 (5.0–26.5)	15.8[a] ± 4.6
TMP/R	CK	19.8 (8.4–36.8)	19.5[a] ± 8.1	32.8 (3.8–45.1)	32.1[a] ± 7.4	25.9 (1.5–36.8)	26.9[a,c] ± 6.8
	DES	39.3 (19.3–53.6)	38.6[a] ± 7.5	42.4 (25.1–59.4)	42.6[a] ± 5.9	7.9 (1.7–24.5)	8.8[c] ± 5.9

CK—cytokeratin; CMP - rats treated with cyclosporin A, mycophenolate mofetil, and prednisone; CMP/R—rats treated with cyclosporin A, mycophenolate mofetil, and prednisone in the first three months and rapamycin in the last three months; DES—desmin; RCP—rats treated with rapamycin, cyclosporin A, and prednisone; RMP—rats treated with rapamycin, mycophenolate mofetil, and prednisone; RTP—rats treated with rapamycin, tacrolimus, and prednisone; TMP - rats treated with tacrolimus, mycophenolate mofetil, and prednisone; TMP/R—rats treated with tacrolimus, mycophenolate mofetil, and prednisone in the first three months and rapamycin in the last three months; x ± SD - arithmetical mean ± standard deviation; a—$p < 0.001$ vs. control; b—$p < 0.05$ vs. CMP; c—$p < 0.05$ vs. TMP (Kruskal-Wallis test, $n = 30$ for each grade of expression in each group).
